# Altered Patterns of the Fractional Amplitude of Low-Frequency Fluctuation in Drug-Naive First-Episode Unipolar and Bipolar Depression

**DOI:** 10.3389/fpsyt.2020.587803

**Published:** 2020-11-17

**Authors:** Xue Chai, Rongrong Zhang, Chen Xue, Zonghong Li, Wang Xiao, Qingling Huang, Chaoyong Xiao, Shiping Xie

**Affiliations:** ^1^Department of Radiology, The Affiliated Brain Hospital of Nanjing Medical University, Nanjing, China; ^2^Department of Psychiatry, The Affiliated Brain Hospital of Nanjing Medical University, Nanjing, China

**Keywords:** bipolar disorder, unipolar depression, amplitude of low-frequency fluctuations, fractional amplitude low-frequency fluctuations, resting-state functional magnetic resonance imaging

## Abstract

**Background:** An early and correct diagnosis is crucial for treatment of unipolar depression (UD) and bipolar disorder (BD). The fractional amplitude of low-frequency fluctuations (fALFFs) has been widely used in the study of neuropsychiatric diseases, as it can detect spontaneous brain activities. This study was conducted to survey changes of fALFF within various frequency bands of the UD and BD patients, as well as to explore the effects on changes in fALFF on cognitive function.

**Methods:** In total, 58 drug-naive first-episode patients, including 32 UD and 26 BD, were enrolled in the study. The fALFF values were calculated under slow-5 band (0.01–0.027 Hz) and slow-4 band (0.027–0.073 Hz) among UD patients, BD patients, and healthy control (HC). Additionally, we conducted correlation analyses to examine the association between altered fALFF values and cognitive function.

**Results:** Under the slow-5 band, compared to the HC group, the UD group showed increased fALFF values in the right cerebellum posterior lobe, whereas the BD group showed increased fALFF values in the left middle temporal gyrus (MTG). Under the slow-4 band, in comparison to HC, the UD group showed increased fALFF values in the left superior temporal gyrus, whereas the right inferior parietal lobule (IPL) and BD group showed increased fALFF values in the bilateral postcentral gyrus. Notably, compared to BD, the UD group showed increased fALFF values in the right IPL under the slow-4 band. Furthermore, altered fALFF values in the left MTG and the right IPL were significantly positively correlated with Verbal Fluency Test scores.

**Conclusions:** This current study indicated that there were changes in brain activities in the early UD and BD groups, and changes were related to executive function. The fALFF values can serve as potential biomarker to diagnose and differentiate UD and BD patients.

## Introduction

Unipolar depression (UD) and bipolar disorder (BD) are highly disabling diseases that seriously impact the patients' quality of life and are associated with high suicide rates (1, 2). In particular, studies have suggested that anxiety/depression, alexithymia, and extreme sensory processing patterns in psychiatric disorders are risk factors for suicide (3, 4) The distinction between UD and BD depends on whether the patient presents with a manic or hypomanic episode (2, 5, 6). It is almost impossible to correctly diagnose BD patients without a history of mania/hypomania episode (6, 7). Studies have shown that about 60% of BD patients are initially misdiagnosed as UD, and they are treated differently, which can cause serious consequences, including inducing manic episodes and emotional instability. These disabling consequences are often associated with abnormal use of antidepressants (8, 9). Therefore, promoting an understanding of the pathological mechanism of UD and BD will be of great help to diagnosis and treat this disease. According to previous studies, brain activities in the resting state, which reflect the baseline status of the brain, contributes to a better understanding of the pathophysiological features of mental illness, including UD and BD.

The resting-state functional magnetic resonance imaging (fMRI), a non-invasive fMRI technique, has been suggested as a useful way to measure spontaneous brain activities and detect internal changes in brain function that are inherent in pathological condition, including mental disorders such as UD and BD (10). The amplitude of low-frequency fluctuations (ALFFs) and the fractional ALFF (fALFF) are two credible indicators that are used to examine the regional features of low-frequency oscillations (LFOs) (11, 12). The ALFF measures voxel-wise amplitude of LFOs at very low frequencies (typically 0.01–0.08 Hz) and reflects local characteristics of brain oscillatory activities, whereas fALFF is the ALFF of a given frequency band and is part of the sum of amplitudes over an entire frequency range (13). Notably, the fALFF is a modified index of ALFF, which is less likely to produce any noise and is more sensitive and specific to the detection of spontaneous brain activities in comparison to ALFF (13). Convergent evidence suggests that the whole frequency range can be divided into four bands, including slow-5: 0.01–0.027 Hz, slow-4: 0.027–0.073 Hz, slow-3: 0.073–0.198 Hz, and slow-2: 0.198–0.25 Hz, in which the slow-4 and slow-5 bands can sensitively reveal pathology behind neuropsychiatric disorders (13, 14). Previous studies have indicated that ALFF/fALFF values under slow-4 and slow-5 bands are of great significance in the diagnosis of depression, but few studies have assessed its application in the UD and BD patients (14).

Most neuroimaging studies of UD and BD patients have found abnormal neuronal activities, which manifest as abnormal ALFF/fALFF values (15, 16). Chun et al. found that the BD group indicated abnormal ALF values that mainly exist in the prefrontal-limbic networks and associated striatal systems (17). The pediatric BD patients have also shown abnormal ALFF values in the basal ganglia, parietal cortex, and occipital cortex (10). Moreover, ALFF values in the bilateral superior frontal gyrus and left superior temporal gyrus can contribute to differentiate early-onset depression from late-onset depression (18). Notably, studies have shown that suicidal ideation is relevant to the changing resting-state brain activities in major depressive disorder (19). Electroconvulsive therapy, as a quick and effective treatment for severe depression, can relieve symptoms of depression, as well as alter resting-state brain activities (20). Therefore, application of ALFF in depression can play a role in diagnosing depression, judging the period of depression, and even providing a new perspective for selection of therapeutic targets.

In this current study, we calculated fALFF values in the slow-4 and slow-5 bands in drug and treatment-naive, first-episode, and short-illness-duration patients with UD and BD. In addition, correlation analyses were applied to study the relationship between altered fALFF values of UD and BD and cognitive function. We hypothesized the fALFF values were conducive to identification of UD and BD patients, and changed fALFF values affected cognitive function of patients.

## Materials and Methods

### Subjects

In total, 58 drug-naive first-episode patients, including 32 UD and 26 BD patients, were recruited from the inpatient and outpatient clinic at the Affiliated Brain Hospital of Nanjing Medical University, Jiangsu Province, China. The inclusion criteria for UD patients included the following: (1) met the criteria outlined in the fourth edition of the *Diagnostic and Statistical Manual of Mental Disorders, Fourth Edition* (*DSM-IV*) for major depressive episode; (2) had a 17-item Hamilton Depression Rating Scale (HAMD_17_) score ≥17; (3) between the ages of 14 and 45 years; (4) right-handed Han Chinese; and (5) never received psychotropic medication, electroconvulsive therapy, or psychotherapy. The inclusion criteria for BD patients included the following: (1) met the *DSM-IV* criteria for BD; (2) currently in the midst of a depressive episode; (3) Young Mania Rating Scale (YMRS) score <7 and 17-item HAMD score ≥17; (4) between the ages of 14 and 45 years; (5) right-handed Han Chinese; and (6) never received psychotropic medication, electroconvulsive therapy, or psychotherapy. The exclusion criteria for the patients included the following: (1) diagnosis of other mental disorders; (2) a history of severe brain injury, organic brain diseases, or systemic illnesses; (3) alcohol or drug abuse; (4) pregnancy and lactation; and (5) contraindications to MRI scanning. Overall, 30 healthy control (HC) individuals were recruited from the community. The inclusion criteria for HC comprise (1) no history of mental disorders, according to *DSM-IV*; (2) HAMD_17_ score <7; and (3) no family history of mental disorders. All participants and their families provided written informed consent, and the study was granted approval by the Institutional Ethical Committee for Clinical Research of Nanjing Brain Hospital.

### Neurocognitive Assessments

The study adopted the Chinese version of the MATRICS Consensus Cognitive Battery (MCCB) in order to assess cognitive function. The MCCB is a comprehensive and systematic assessment that can be divided into seven domains: (1) speed of processing: Trail Making Test A, Brief Assessment of Cognition in Schizophrenia, and Semantic Verbal Fluency Test (VFT); (2) verbal learning: Hopkins Vocabulary Learning Test; (3) working memory: Wechsler Memory Scale; (4) reasoning and problem-solving: Neuropsychological Assessment Battery (NAB); (5) visual learning: the Brief Visuospatial Memory Test; (6) social cognitive: Mayer–Salovey–Caruso Emotional Intelligence Test; and (7) attention/vigilance: Continuous Performance Test, Identical Pairs version (CPT-IP) (21).

### MRI Data Acquisition

Neuroimaging was conducted through the use of a 3.0-T Verio Siemens scanner with an eight-channel head-coil at the Nanjing Brain Hospital. Participants were instructed to rest with their eyes open, to not fall asleep, and to not think about anything in particular.

The gradient-echo echoplanar imaging sequence contained 149 volumes. The parameters included repetition time (TR) = 2,500 ms, echo time (TE) = 30 ms, flip angle = 90°, slice thickness = 3.5 mm, number of slices = 37, field of view (FOV) = 224 × 224 mm^2^, matrix size = 64 × 64, and voxel size = 3.5 × 3.5 × 3.5 mm^3^.

High-resolution T1-weighted images were obtained using magnetization-prepared rapid gradient-echo sequence. The parameters included TR = 2,300 ms, TE = 2.98 ms, flip angle = 9°, inversion time = 900 ms, thickness = 1 mm, number of slices = 192, FOV = 256 × 240 mm^2^, and voxel size = 1 × 1 × 1 mm^3^.

### Image Preprocessing

The fMRI data were preprocessed using Data Processing and Analysis for Brain Imaging (DPABI, http://rfmri.org/DPABI) software in MATLAB 2013b (http://www.mathworks.com/products/matlab/). The first five volumes of functional images were discarded, and the remaining 142 volumes were used for subsequent processing, including slice timing, head-motion correction, and nuisance covariate regression. There were no subjects with excessive head motion (>2.5 mm of displacement or >2.5° of rotation in any direction). Next, 24 motion parameters, global signal, white matter signal, and cerebrospinal fluid signal were chosen as nuisance covariate. Then, the functional images were spatially normalized to the standard space of the Montreal Neurological Institute and resampled to an isotropic voxel size of 3 mm. Finally, the resulting functional images were smoothed with a 6-mm full-width half-maximum Gaussian kernel, and the linear trends were removed.

### fALFF Measurement

The fALFF computed using the DPABI software. The time series of each voxel was transformed into a frequency domain, and the power spectrum was obtained using the fast Fourier transform. The square root was measured at each frequency of the power spectrum, and the averaged square root, i.e., ALFF value, was acquired over the range of 0.01–0.08 Hz. The fALFF value was obtained by dividing total ALFF values from 0.01 to 0.025 Hz. Finally, the resulting fALFF maps were normalized with each voxel divided by mean of the fALFF values of the whole-brain signal, providing mfALFF spatial maps. In addition, this study calculated and analyzed fALFF at slow-4 band (0.027–0.073) and slow-5 band (0.01–0.027), according to previous studies.

### Statistical Analysis

The Statistical Package for the Social Sciences software version 22.0 (IBM, Armonk, New York) was used for statistical analysis. The analysis of variance (ANOVA), the two-sample *t*-test, and χ^2^-test were performed compared with the demographics and neurocognition among the groups, including UD, BD, and HC. The threshold for statistical significance difference was set at *p* < 0.05.

One-way ANOVA was conducted in order to compare differences in mfALFF value between the three groups, including UD, BD, and HC using age, gender, and education as covariates, Non-parametric permutation test was conducted in the study in order to reduce the false-positive rate, and the permutation times were set at 1,000. *p* < 0.05 was set for statistical significance, and cluster size >200 voxels (5,400 mm^3^) was used for multiple comparisons. The *post-hoc* comparisons were calculated using a mask obtained from ANOVA using age, gender, and education as covariates. The non-parametric threshold-free cluster enhancement (TFCE) and the family-wise error (FWE) corrected at *p* < 0.05 with cluster size >20 voxels (540 mm^3^) were set for statistical significance. The mfALFF values of significantly changed regions were extracted using the Resting-State fMRI Data Analysis Toolkit (Rest) (http://www.restfmri.net/forum/REST_V1.8) and used for correlation analysis. The correlation analysis was conducted to reveal the relationships between altered mfALFF values and MCCB assessments with age, gender, and years of education as covariates (*p* < 0.05, Bonferroni-corrected).

## Results

### Demographic and Neurocognitive Characteristics

The demographic and MCCB information of all subjects, including 32 UD, 26 BD, and 30 HC patients, is shown in Table 1. The results indicated significant differences in NAB, VFT, and CPT-IP across the three groups. In comparison to HC, both UD and BD showed significantly reduced NAB and VFT scores, whereas only BD showed significantly reduced CPT-IP (*p* < 0.05). All results were obtained using age, gender, and education as covariates.

**Table 1:** Demographics and MCCB assessments of patients with UD, BD, and HC subjects.

	**UD (32)**	**BD (26)**	**HC (30)**	***F*/χ^**2**^**	***p***
Age (years)	26.2 (7.0)	22.7 (5.7)	22.5 (4.0)	4.023	0.021
Gender (male/female)	17/15	10/16	12/18	1.594	0.451
Education level (years)	14.6 (2.6)	13.8 (2.4)	14.4 (3.1)	0.939	0.395
Course of disease (month)	18.5 (22.9)	25.0 (20.7)	—	−1.060	0.294
TMT	31.5 (8.4)	33.04 (10.2)	27.3 (6.6)	2.754	0.070
BACS	62.7 (11.1)	61.1 (10.2)	66.8 (4.4)	2.436	0.094
HVLT-R	27.8 (4.7)	27.8 (4.7)	28.2 (3.2)	0.083	0.920
WMS	16.9 (3.6)	16.3 (2.8)	17.7 (1.7)	1.256	0.291
NAB	17.5 (7.2)*	17.7 (5.7)*	22.0 (3.2)	4.331	0.016
BVMT-R	27.4 (4.9)	27.9 (5.0)	29.1 (3.6)	0.387	0.680
VFT	22.2 (5.9)*	22.3 (3.8)*	25.9 (4.1)	4.666	0.012
MSCEIT	86.6 (8.5)	87.5 (9.7)	89.1 (6.3)	0.408	0.666
CPT-IP	2.8 (0.6)	2.5(0.6)*	3.0 (0.4)	5.097	0.008

Numbers are given as means (standard deviation) unless stated otherwise. Significant group differences were found at p < 0.05 (ANOVA test and post-hoc analysis). *Compared to HC. UD, unipolar depression; BD, bipolar disorder; HC, healthy control; TMT, Trail Making Test; BACS, Brief Assessment of Cognition in Schizophrenia; HVLT-R, Hopkins Vocabulary Learning Test; WMS, Wechsler Memory Scale; NAB, Neuropsychological Assessment Battery; BVMT-R, the Brief Visuospatial Memory Test; VFT, Verbal Fluency Test; MSCEIT, Mayer–Salovey–Caruso Emotional Intelligence Test; CPT-IP, Continuous Performance Test, Identical Pairs version.

### Altered mfALFF Values in the Three Groups

**Figure 1:**
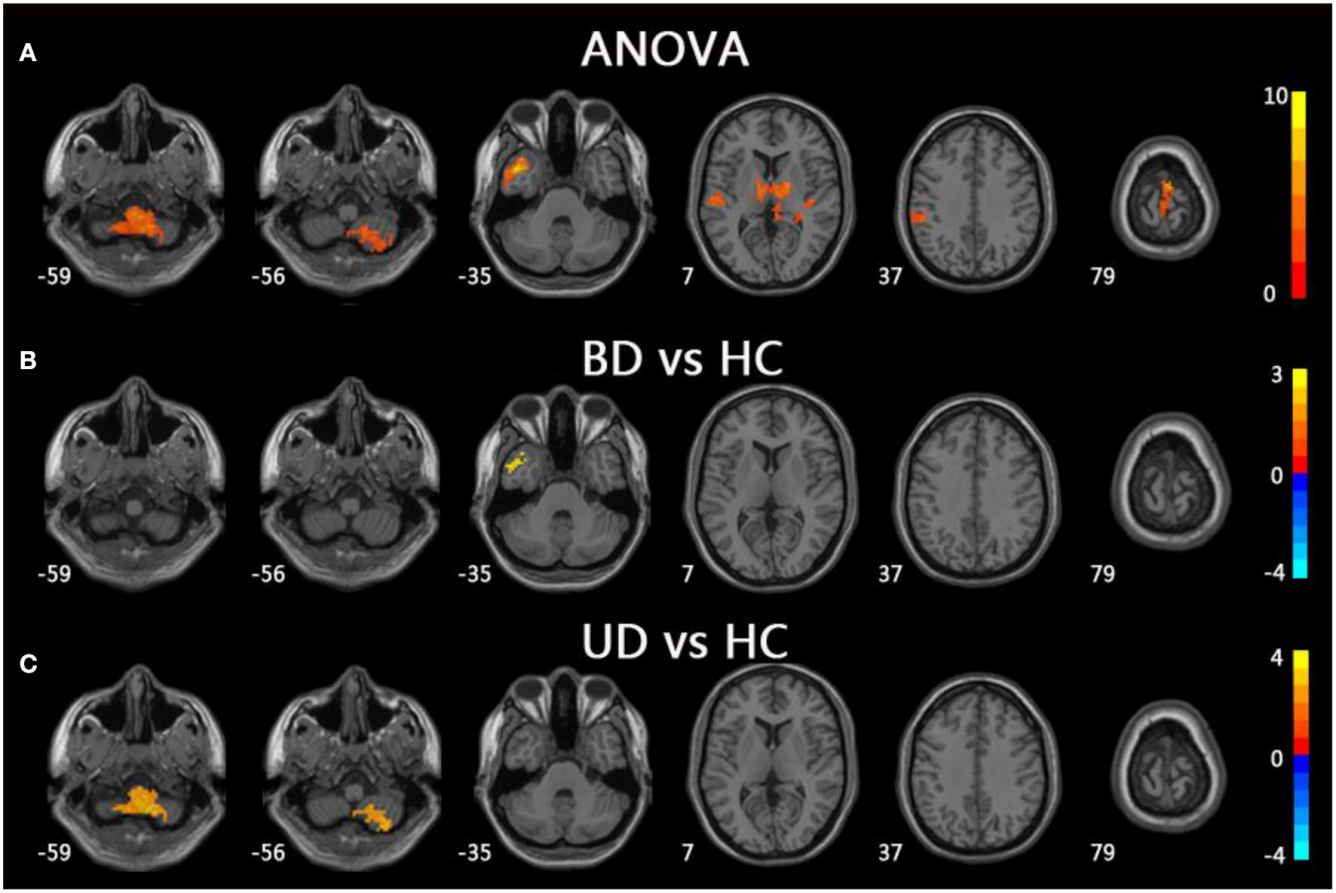
Brain regions exhibiting significant differences from ANOVA and *post-hoc* analysis in fALFF values under slow-5 band. **(A)** Brain regions showing significant differences in fALFF values between UD, BD, and HC (cluster size >200, *p* < 0.05). **(B)** Brain regions showing significant differences in fALFF values between BD and HC (cluster size >19, TFCE-FWE corrected, *p* < 0.05). **(C)** Brain regions showing significant differences in fALFF values between UD and HC (cluster size > 19, TFCE-FWE corrected, *p* < 0.05). UD, unipolar depression; BD, bipolar disorder; HC, healthy control.

For mfALFF values in slow-5, the ANOVA indicated significant differences across the three groups, including UD, BD, and HC, and included the right cerebellum posterior lobe (CPL), the left middle temporal gyrus (MTG), the bilateral thalamus, the left inferior parietal lobule (IPL), and the left precentral gyrus. Compared to HC, the BD group showed significantly increased mfALFF value in the left MTG, whereas the UD group demonstrated increased mfALFF value in right CPL (TFCE-FWE corrected, cluster size ≥20, *p* < 0.05). All results were obtained using age, gender, and education as covariates (Table 2, Figure 1).

**Table 2:** The difference of fALFF values under slow-5 band across three groups.

**Brain region**	**Peak MNI coordinate**			***F*/*t***	**Cluster number**
	***x***	***y***	***z***		
**ANOVA**					
R cerebellum posterior lobe	6	−42	−63	10.4337	225
L middle temporal gyrus/superior temporal gyrus	−42	9	−36	11.2087	223
B thalamus	18	−6	9	9.8102	572
L inferior parietal lobule/postcentral gyrus	−60	−18	21	7.1453	244
L precentral gyrus	6	−9	78	7.8028	202
**BD vs. HC**					
L middle temporal gyrus	−45	9	−36	3.976	23
**UD vs. HC**					
R cerebellum posterior lobe	9	−42	−63	4.6847	211

*The x, y, and z coordinates is the primary peak locations in the MNI space. Cluster size >200 voxels in ANOVA, p < 0.05; cluster size >19 voxels in post-hoc analysis, TFCE-FEW corrected, p < 0.05. L, left; R, right*.

**Figure 2:**
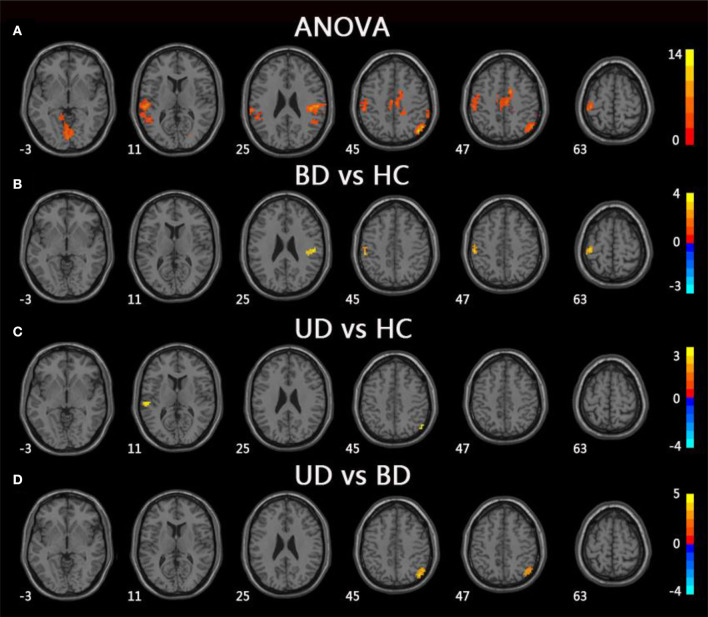
Brain regions exhibiting significant differences from ANOVA and *post-hoc* analysis in fALFF values under slow-4 band. **(A)** Brain regions showing significant differences in fALFF values between UD, BD, and HC (cluster size >200, *p* < 0.05). **(B)** Brain regions showing significant differences in fALFF values between BD and HC (cluster size >19, TFCE-FWE corrected, *p* < 0.05). **(C)** Brain regions showing significant differences in fALFF values between UD and HC (cluster size >19, TFCE-FWE corrected, *p* < 0.05). **(D)** Brain regions showing significant differences in fALFF values between UD and BD (cluster size >19, TFCE-FWE corrected, *p* < 0.05). UD, unipolar depression; BD, bipolar disorder; HC, healthy control.

For mfALFF values in slow-4, ANOVA showed significant differences across three groups, including UD, BD, and HC, as well as the bilateral lingual, the left superior temporal gyrus (STG), the left postcentral gyrus (PoCG), the right IPL, the right PoCG, and the bilateral middle cingulum gyrus. Compared to the HC group, the BD group showed significantly increased mfALFF values in the bilateral PoCG, whereas the UD group showed significantly increased mfALFF values in the left STG and right IPL. Notably, in comparison to the BD group, the UD group showed increased mfALFF values in the right IPL (TFCE-FWE corrected, cluster size ≥20, *p* < 0.05). All results were obtained using age, gender, and education as covariates (Table 3, Figure 2).

**Table 3:** The difference of fALFF values under slow-4 band across three groups.

**Brain region**	**Peak MNI coordinate**			***F*/*t***	**Cluster number**
	***x***	***y***	***z***		
**ANOVA**					
B lingual	−3	−81	−6	8.0345	238
L superior temporal gyrus/postcentral gyrus/inferior parietal lobule	−63	−27	27	9.5035	549
R inferior parietal lobule/angular/postcentral gyrus	45	−69	45	12.5965	419
B cingulum_mid/medial frontal gyrus	12	−27	36	9.9791	357
**BD vs. HC**					
R postcentral gyrus	51	−21	24	4.1787	26
L postcentral gyrus	−42	−27	63	4.3138	89
**UD vs. HC**					
L superior temporal gyrus	−57	−27	12	3.9197	22
R Inferior Parietal Lobule	51	−63	42	3.6236	20
**UD vs. BD**					
R inferior parietal lobule	45	−72	45	5.2864	78

*The x, y, and z coordinates is the primary peak locations in the MNI space. Cluster size >200 voxels in ANOVA, p < 0.05; cluster size >19 voxels in post-hoc analysis, TFCE-FEW corrected, p < 0.05. L, left; R, right*.

### Correlations Between the Abnormal mfALFF Values and MCCB in the Three Groups

**Figure 3:**
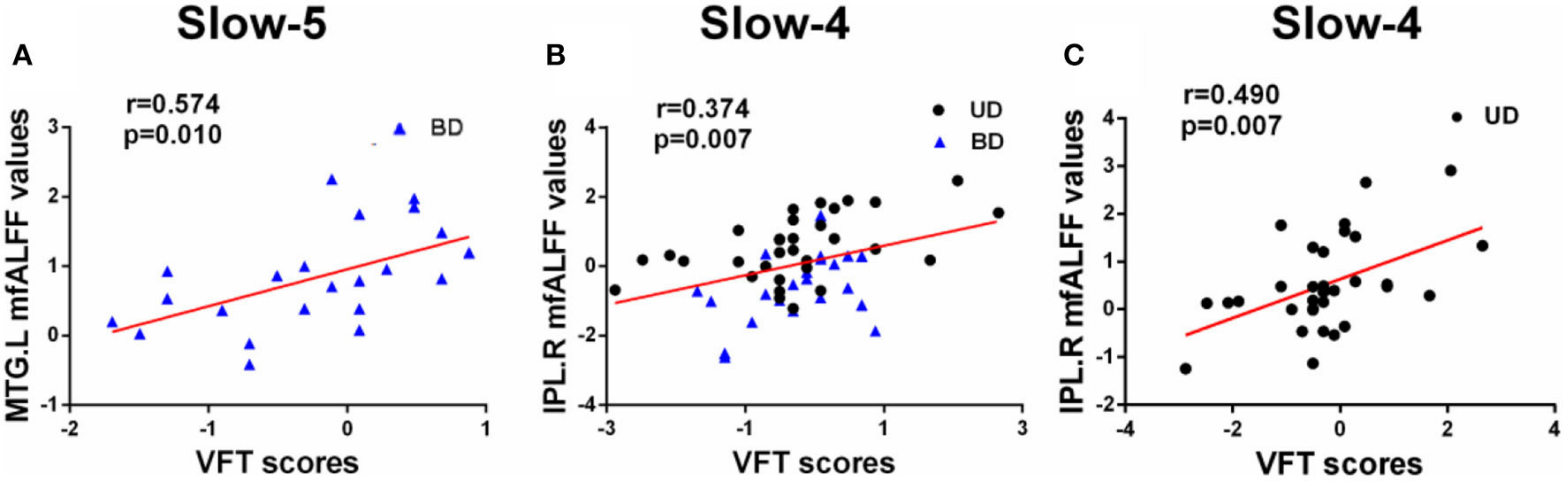
**(A–C)** Significant correlations between altered fALFF values under slow-5 and slow-4 bands and MCCB assessments (Bonferroni corrected, *p* < 0.05). UD, unipolar depression; BD, bipolar disorder; VFT, Verbal Fluency Test; MTG, middle temporal gyrus; IPL, inferior parietal lobule; R, right; L, left.

Pearson correlation analysis was performed between altered mfALFF-values regions and MCCB assessments using age, gender, and years of education as covariates (*p* < 0.05, Bonferroni corrected). Under the slow-5 band, mfALFF values of the left MTG were significantly positively correlated with VFT scores in the BD and HC groups (*r* = 0.574, *p* = 0.010). Under the slow-4 band, the mfALFF values of the right IPL were significantly positively correlated with VFT scores in the groups of BD and MD (*r* = 0.374, *p* = 0.007) and the mfALFF values of the right IPL were positively associated with VFT scores in the MD and HC groups (*r* = 0.490, *p* = 0.007) (Figure 3).

## Discussion

This current study analyzed fALFF values under the slow-4 (0.027–0.073) and slow-5 (0.01–0.027 Hz) bands in BD and UD patients and further explored the relationship between altered fALFF values and MCCB assessments. Our study had several findings that were highly congruent with our hypothesis. First, local brain regions, including the left MTG, the right CPL, the bilateral PoCG, the left STG, and the right IPL, showed significantly increased fALFF under the two separate frequency bands of the patient group. Second, under the slow-4 band, the fALFF values of the right IPL served as a potential biological marker in order to identify the BD and UD groups. Finally, Pearson correlation analysis proved that regions with altered fALFF were positively correlated with impaired VFT scores. In conclusion, the present study revealed that the fALFF values under slow-4 and slow-5 bands play an important and different role in the identification and diagnosis of the very early BD and UD.

### Increased fALFF Values in the BD and UD Groups Under Slow-4 and Slow-5 Bands

Our study indicated that several regions of the BD and UD groups had significantly increased fALFF values in the slow-4 and slow-5 bands. In general, abnormal fALFF values in early BD and UD patients were mainly located in the temporal and parietal lobes, similar to previous studies (22–24).

Under the slow-5 band, compared to the HC group, BD patients showed increased fALFF values in the left MTG, whereas UD patients showed increased fALFF values in the right CPL. Evidence also suggested that BD patients had structural and functional abnormalities in the temporal lobe and that increased fALFF values in the left MTG were consistent with the results of previous studies (15, 25). The cerebellum was thought to be involved in motor processing. However, a growing body of research suggests that the cerebellum is involved in pathogenesis of depression and CPL, which belongs to the default mode network (DMN) and regulates emotional processing and cognition (26). Sun et al. found that both major depressive disorder and cognitive vulnerability to depression showed increased regional homogeneity (ReHo) compared to the HC group, and the abnormal ReHo values were associated with the Center for Epidemiologic Studies Depression Scale (27). Additionally, the decreased functional connection in the bilateral CPL, as well as decreased cerebellar volume, was reported in UD patients in prior studies (28). In combination with increased fALFF values in UD patients of the study, our data suggest that the CPL abnormal resting-state brain activities of CPL may be potential biomarkers of UD patients (29, 30).

Under the slow-4 bands, compared to the HC group, the BD group showed increased fALFF values in the bilateral PoCG, whereas the UD group showed increased fALFF values in the left STG and right IPL. The PoCG, part of the parietal lobe, is widely known to be an important region that is responsible for proprioception (31). A growing body of study suggests that the PoCG is important in emotional processing, including the production of an emotional state and regulation of emotion (31). Abnormalities in the emotional processing circuit in BD patients are related to abnormal brain activities in the PoCG. Moreover, numerous studies have discovered that STG is the key brain region for pathological development of BD patients (32–34). An abnormal fatty acid pattern in STG was even believed to be the specific change in BD patients (35). Interestingly, results from this study showed that fALFF values were increased in the UD group, which is consistent with a meta-analysis, suggesting that both BD and UD patients had functional changes in STG (36). Notably, compared to the BD group, the UD group showed increased fALFF values in the right IPL. The IPL, which is widely considered to be the posterior region of the DMN, is a heterogeneous area that integrates information from different sensory patterns (37, 38). Moreover, the IPL was thought to be involved in cognitive functions, including executive control and episodic memory (39). Previous studies have reported that the UD group showed significantly increased ReHo in the right IPL compared to the HC group. Additionally, Sawamura et al. found that the average kurtosis of gray matter in the right IPL can help distinguish BD from major depressive disorder with a high accuracy (40, 41). It is worth noting that there were no significant differences with regard to the fALFF values of right IPL between BD patients and the HC group, and there was significantly increased fALFF of right IPL in the UD group compared to the HC group. This might suggest the fALFF of right IPL is a specific indicator that can be used to diagnose and identify UD patients.

It is worth noting that there is no reduction in fALFF values of the BD and UD groups in either the slow-4 or slow-5 band, which is clearly inconsistent with prior studies. The reason may be that the patients enrolled in this study were first-episode, drug-naive patients and had a short illness duration and thus belonged to a very early stage of depression and had not yet progressed to a decline in fALFF values. The increased fALFF values might be a compensatory mechanism for early BD and UD. In conclusion, the fALFF values under slow-4 and slow-5 bands can reveal the difference in brain activities between the depression patients and HC. Compared to the slow-5 band, the slow-4 band was more sensitive to differences in brain activities between the BD and UD groups. The combination of the slow-4 and slow-5 bands is beneficial to a clinical diagnosis and differentiation of UD and BD patients.

### Behavioral Significance of Altered fALFF in Three Groups

The current study showed a significantly positive relationship between altered fALFF values and VFT scores in UD and BD patients. Under the slow-5 band, increased fALFF values of the MTG in BD patients were obviously positively correlated with VFT scores. Under the slow-4 band, increased fALFF values of the right IPL in BD and UD patient groups were positively correlated with VFT scores, and increased fALFF values of the right IPL in UD patients were positively correlated to VFT scores. The VFT scale is an important scale that can effectively reflect executive function. The IPL is considered to support control processes, and IPL activity is related to performance accuracy (42, 43). Notably, MTG and IPL are part of the DMN, which is deemed to be involved in self-referential processing, including internal monitoring, memory retrieval, theory of mind, and future planning (44, 45). Therefore, the left MTG and right IPL are regarded to play vital roles in executive function in early BD and UD patients.

## Limitations

There are two limitations in the current study that should be mentioned. First, the small sample size in the study may influence the authenticity of results. Therefore, we will continue to recruit volunteers and continue the analysis. Second, there were significant differences in age among the three groups, which may have influenced the results. To solve this problem, we performed all statistical analyses using age, gender, and years of education as covariates.

## Conclusion

Distinctively abnormal fALFF values were found among three groups, including UD, BD, and HC under the slow-4 and slow-5 bands, which revealed varying degrees of altered brain activities in UD and BD patients. In comparison to slow-5 bands, the slow-4 bands were more sensitive to differences in brain activities between the BD and UD groups. Moreover, the positive correlation between altered fALFF values and VFT scores further suggested that abnormal fALFF may contribute to a cognitive decline in UD and BD patients. In summary, the fALFF values can be used as a biological marker in the BD and UD patients.

## Data Availability Statement

The raw data supporting the conclusions of this article will be made available by the authors, without undue reservation.

## Ethics Statement

The studies involving human participants were reviewed and approved by the Institutional Ethical Committee for clinical research of Nanjing Brain Hospital. Written informed consent to participate in this study was provided by the participants' legal guardian/next of kin.

## Author Contributions

XC, RZ, CXi, and SX: designed the study. XC, RZ, CXu, ZL, WX, QH, CXi, and SX: collected the data. XC and RZ: analyzed the data and prepared the manuscript. All authors contributed to the article and approved the submitted version.

## Conflict of Interest

The authors declare that the research was conducted in the absence of any commercial or financial relationships that could be construed as a potential conflict of interest.
